# Cell Wall Polymer Composition and Spatial Distribution in Ripe Banana and Mango Fruit: Implications for Cell Adhesion and Texture Perception

**DOI:** 10.3389/fpls.2019.00858

**Published:** 2019-07-09

**Authors:** Ganittha Rongkaumpan, Sam Amsbury, Efren Andablo-Reyes, Holly Linford, Simon Connell, J. Paul Knox, Anwesha Sarkar, Yoselin Benitez-Alfonso, Caroline Orfila

**Affiliations:** ^1^Nutritional Sciences and Epidemiology Group, School of Food Science and Nutrition, University of Leeds, Leeds, United Kingdom; ^2^Centre for Plant Sciences, Faculty of Biological Sciences, University of Leeds, Leeds, United Kingdom; ^3^Food Colloids and Bioprocessing, School of Food Science and Nutrition, University of Leeds, Leeds, United Kingdom; ^4^School of Physics and Astronomy, University of Leeds, Leeds, United Kingdom

**Keywords:** cell wall, banana, mango, texture, hemicellulose, pectin, cell adhesion, tribology

## Abstract

Banana (*Musa acuminata*) and mango (*Mangifera indica*) are two of the most popular fruits eaten worldwide. They both soften during ripening but their textural attributes are markedly different. This study aimed to elucidate the molecular mechanism underpinning textural differences between banana and mango. We used a novel combination of methods at different scales to analyse the surface properties of fruit cells and the potential contribution of cells and cell wall components to oral processing and texture perception. The results indicated that cell separation occurred easily in both organs under mild mechanical stress. Banana cells showed distinctively elongated shapes with distinct distribution of pectin and hemicellulose epitopes at the cell surface. In contrast, mango had relatively spherical cells that ruptured during cell separation. Atomic force microscopy detected soft surfaces indicative of middle lamella remnants on banana cells, while mango cells had cleaner, smoother surfaces, suggesting absence of middle lamellae and more advanced cell wall disassembly. Comparison of solubilized polymers by cell wall glycome analysis showed abundance of mannan and feruylated xylan in separation exudate from banana but not mango, but comparable levels of pectin and arabinogalactan proteins. Bulk rheology experiments showed that both fruits had similar apparent viscosity and hence might be extrapolated to have similar “oral thickness” perception. On the other hand, oral tribology experiments showed significant differences in their frictional behavior at orally relevant speeds. The instrumental lubrication behavior can be interpreted as “smooth” mouthfeel for mango as compared to “astringent” or “dry” for banana in the later stages of oral processing. The results suggest that cell wall surface properties contribute to lubricating behavior associated with textural perception in the oral phase.

## Introduction

Banana (*Musa acuminata*) and mango (*Mangifera indica*) are two important tropical crops consumed worldwide for their sensorial and nutritional attributes. However, their texture at the ripe stage are markedly different. Textural perception of fruits is determined through complex signals including the physical and chemical responses to food components. Texture is the second most important aspect for sensorial acceptability of fleshy fruit besides visual appearance (Contador et al., 2015). Although sensory analysis and rheological testing are the classical approaches to determine textural perception (Colin-Henrion et al., 2007; Charles et al., 2017), it is lately claimed that oral processing involves not only bulk rheology (e.g., viscosity) but also surface-dominated tribological (e.g., friction and lubrication) phenomena particularly at the later stages of oral processing (Chen and Stokes, 2012; Stokes et al., 2013; Sarkar et al., 2019). Recently, tribology has been successfully employed to understand surface-dominated oral perception using empirical correlations between friction coefficients (μ) and mouthfeel attributes, such as slipperiness and pastiness for biopolymeric hydrogels (Krop et al., 2019). To date, tribological measurements have not been employed to quantitatively understand the mechanisms behind the textural perception of fruits and fruit cells. The importance of solid content and particle size on rheological and sensory properties of fruit purees and suspension has been previously explored, particularly in apple (Espinosa et al., 2011). However, the role of cell adhesion and the effect of intact cells or cell wall ghosts on oral perception is still not clearly understood.

Both banana and mango have been described as having a “melting texture” in which the tissue disintegrates in the oral cavity without chewing (Contador et al., 2015). Ripe banana fruit elicit a complex textural response, described as mealy and slightly astringent texture (Valente et al., 2011) which contrasts with the fleshy, slippery and juicy texture of mango fruit (Suwonsichon et al., 2012). Both types of fruit undergo climacteric ripening with rapid biochemical and biophysical changes resulting in fruit softening within a few days of ripening onset (Ali et al., 2004). Several coordinated processes lead to the disassembly of the cell wall and middle lamellae, resulting in loss of turgor and cell separation (Brummell and Harpster, 2001). Cell wall disassembly has been extensively studied in tomato (*Solanum esculentum*) as a model system of climacteric fruit ripening (Rose and Bennett, 1999; Wang et al., 2018). Even though banana has been suggested as a model system for ripening of monocotyledonous plants (D’Hont et al., 2012), little is known about how the banana cell wall disassembles. Strong up- regulation of genes (up to 12-fold) encoding pectin lyases (PL), xyloglucan endotransglycosylase/hydrolases (XTH) and expansins was observed in ripe fruit compared to unripe fruit, while some isoforms endo-polygalacturonase (PG), pectin methyl esterase (PME) and cellulase were also up-regulated to a lesser extent (Asif et al., 2014). In mango (a dicotyledonous species), several cell wall modifying enzymes have been found to be expressed during ripening, including PL (Chourasia et al., 2006), endo-PG (Chourasia et al., 2006) and beta-glucanase (Chourasia et al., 2008). Mango fruit have a similar melting texture to persimmon (*Diospyros kaki* L.) where several XTH isoforms were suggested to be involved in cell wall remodeling leading to softening (Han et al., 2015). Cell wall enzyme activities are thought to increase solubility of pectins and hemicelluloses (Muda et al., 1995; Prado et al., 2016), possibly through a debranching process that decreases polymer interactions (Posé et al., 2018). How these activities occur in space and time during the ripening of different fruits, and how they contribute to texture and oral perception, is not clearly understood.

Moreover, the role of cell adhesion and specific cell wall polymers on oral processing and texture perception are still poorly understood. It is worth noting that some cell wall enzymes continue to be active in the oral phase and their activities may influence texture. In tomato, PME activity was detected in simulated oral processing conditions and was associated with decreased viscosity within 1 min of oral processing time (Rabiti et al., 2018). Furthermore, the intactness of fruit cell walls is a strong positive determinant of the viscosity of fruit products (Chu et al., 2017) and negatively associated with fermentation potential by microbiota (Low et al., 2015). Both these properties are important for the health benefits associated with fruit intake (Dreher, 2018).

Visualization of cell wall polymers *in muro* using antibody probes can provide insight to polymer function (Lee et al., 2011), and this approach suggested a potential role for different pectin and xyloglucan domains in mediating cell adhesion in ripening tomato fruit (Orfila et al., 2001; Ordaz-Ortiz et al., 2009). Antibodies are also useful tools to profile polysaccharide epitopes within polysaccharide populations extracted from cell walls (Pattathil et al., 2010; Cornuault et al., 2014), although this technique has not been previously used to evaluate polymers solubilized during cell separation. Atomic force microscopy has been used to visualize the structure of cell wall fractions from fruits (Paniagua et al., 2014; Cárdenas-Pérez et al., 2018; Posé et al., 2018) and intact cell surface of onion cells (Zhang et al., 2016). AFM provides additional structural information to immunofluorescence microscopy.

This study aimed to elucidate the molecular mechanism underpinning textural differences between banana and mango. We used a novel combination of methods at different scales to analyse the properties of separated fruit cells and their potential contribution to oral processing and texture perception.

## Materials and Methods

### Plant Materials

Banana (*Musa acuminata* var Cavendish) and mango (*Mangifera indica* var Kesar) fruits were purchased in a market in Leeds, England. Mango fruits were classed at stage five, were soft and fully ripe without any signs of decay (Nambi et al., 2015). Banana fruit were at stage seven with yellow color, soft texture and brown spots (Soltani et al., 2010). Fruits were peeled and parenchyma tissue was gently scraped using a metal spatula, passed through a large-mesh sieve (250 μm) and transferred to a test tube containing MiIliQ water to a final suspension of 9.0 wt%. A sample of supernatant was collected for the glycome analysis of solubilized polymers. Two fruit from each species were processed as biological replicates for each experiment. Representative photographs were chosen for labeling and AFM experiments.

### Bulk Rheology

Rheological characterization of the mango or banana cell suspensions (9.0 wt% cell in MiIliQ water) was conducted using a controlled-stress rheometer (Kinexus Ultra, Malvern Instruments Ltd, Worcestershire, United Kingdom). Temperature was controlled at 37°C to mimic the physiological conditions. A cone-on-plate geometry (40 mm, 4°) was used to measure the steady state flow behavior as a function of shear rate ranging from 0.1 to 1000 s^–1^. Results are presented as means and standard deviations of at least three measurements of each fruit suspension sample. Two fruit from each species were processed as biological replicates.

### Soft Tribology

Friction measurements were performed in presence of cell suspensions (9.0 wt% mango or banana cells in MilliQ water) using a Mini Traction Machine 2 (MTM2, PCS instruments, London, United Kingdom) with a soft polymeric ball-on-disc set up using slight modification of the previously described method (Laguna et al., 2017; Krop et al., 2019). The tribological set up included hydrophobic contact surfaces (water contact angle of 108^∘^ (Sarkar et al., 2017) involving a smooth polydimethylsiloxane (PDMS) ball (6.35 mm radius) on smooth PDMS disc (13 mm radius, 4 mm thick) within a mini-pot chamber. A fresh ball and disc was used for each individual measurement and all friction measurements were carried out at 37°C to mimic oral conditions. A normal load (*F*_*n*_) of 2 N was used in all experiments and the entrainment speeds were varied from 300 to 3 mm s^–1^. The entrainment speed (*U*) was calculated using equation (1):

(1) $$U=\frac{1}{2}\,\left(U_{B}+U_{D}\right)$$

Where, *U*_*B*_ and *U*_*D*_ are the speeds of the ball and disc, respectively. The slide-to-roll ratio defined as |*U*_*B*_−*U*_*D*_|/*U* was fixed at 50%. The friction force (*F_*f*_* = μ.*F*_*n*_) was measured as a function of entrainment speeds and the dimensionless friction coefficient (μ) was reported as means and standard deviations of at least three measurements of each fruit suspension sample. Two fruit from each species were processed as biological replicates.

### Cell Surface Cytochemical Staining

For non-specific staining of cell membrane and contents, 0.05% (w/v) Toluidine Blue O (T3260, Sigma-Aldrich) in 0.1 M phosphate buffer pH 6.8 was added to the fruit tissue in the tube. After staining for 5 min, the stained samples were mounted onto poly-L-lysine coated slides (Polysine, J2800AMNZ, Thermo-Scientific). For starch staining, the fruit tissue was dispersed in distilled water and placed on a polysine coated slide, then one drop of Gram’s iodine solution (90107, Sigma-Aldrich) was added and mixed directly on the slide. For cellulose staining, 0.1% (w/v) Calcofluor White stain [Fluorescent Brightener 28 (319945), Sigma-Aldrich] was added to fruit tissue in the tube. One drop of stained sample was placed on a polysine coated slide, then made alkaline with one drop of 10% (v/v) NaOH. The sample were observed using an inverted light microscope for Toluidine Blue O and iodine staining, and UV fluorescence microscope for Calcofluor White staining (Olympus, model BH2, Japan). Images were captured using a digital camera (Sony, model sCMEX-3). All staining was done at room temperature.

### Cell Surface Immunofluorescence Labeling

Fruit tissue was collected as described above. The surface of fruit cells were immunolabeled with rat monoclonal antibodies to plant cell wall polysaccharide epitopes. Seven antibodies were selected for this experiment: LM28 (Cornuault et al., 2015), LM25 (Pedersen et al., 2012), LM21 (Marcus et al., 2010), JIM5 and JIM7 (Clausen et al., 2003), LM5 (Jones et al., 1997), LM6-M (Cornuault et al., 2017). A list of antibodies and the epitopes is available at http://www.plants.leeds.ac.uk/pk/pdf/JPKab05.pdf. Antibody hybridoma supernatants were diluted 10 times in 3% (w/v) non-fat dry milk (Marvel) in 10 mM phosphate-buffered saline (PBS) before use. Firstly, the silane-prep slides (Thermo-fisher) were activated using 2.5% (v/v) glutaraldehyde (A17876, Sigma-Aldrich, St. Louis, MO, United States) in PBS pH 7.45. Suspended fruit cells (50 μl) were added to an activated silane-prep slide, followed by quick drying for 10 min on a hot plate. Surface non-specific epitopes were blocked with 50 μl of 3% (w/v) non-fat dry milk in 10 mM PBS for 30 min. Subsequently, fruit cells were labeled with selected monoclonal antibodies for 1 h. After washing with PBS three times for 5 min each, the fruit cells were incubated with 100-fold dilution of anti-rat IgG-FITC (F1763, Sigma-Aldrich, St. Louis, MO, United States) in 3% (w/v) non-fat dry milk in 10 mM PBS for 1 h, followed by three 5 min washes with PBS. Citifluor AF1 antifade reagent (AGR1320, Agar Scientific) was added on the slide before examining under fluorescence microscope (Olympus, model BH2) equipped with blue epifluorescence. In terms of negative control, the sample was treated according to the steps described above with omission of primary monoclonal antibody. All labeling steps were done at room temperature.

### Cell Surface Atomic Force Microscopy (AFM)

Fruit tissue was collected as described above. Cell suspensions were further passed through a medium-mesh metal sieve (150 μm) to remove loose starch, with retentate being washed with MilliQ water (3 × 50 mL) and resuspended in MilliQ water. 200 μL of cell suspension was applied to a glass coverslip and allowed to dry for at least 48 h (room temperature) before AFM imaging. Dried samples were imaged using a Multimode^®^ AFM with J scanner (Bruker, CA, United States), with PF QNM (PeakForce Quantitative Nanomechanical Property Mapping). Images were flattened to remove bow in each scan line and exported in TIFF format. At least five different cells were scanned for each sample at 0.8–0.9 Hz. Only whole individual cells were selected for imaging (i.e., cells that were not attached to other cells), minimizing the likelihood that an inner surface would be imaged. Five regions on each cell were chosen in areas that did not cross over an obvious fold or wrinkle caused by drying round cells onto a flat surface. Representative images were then selected for the paper.

### Preparation of Alcohol Insoluble Residue (AIR)

Alcohol insoluble residue from each fruit was prepared. Fruit tissue (3 *g*) was homogenized at 13000 *g* (Polytron model 2500 E, Switzerland) with 7 *g* of 100% ethanol for around 1 min until a homogeneous sample was achieved, giving final ethanol concentration of 70%. Then, the sample was centrifuged at 3500 *g* for 20 min (Heraeus Megafuge 16R centrifuge, Germany) at room temperature. The supernatant was removed, and the residue was resuspended in 70% (v/v) ethanol, homogenized at 13000 *g* for 30 s and centrifuged at 5000 rpm for 20 min. The residue was repeatedly washed with a series of solvents: 80% (v/v) ethanol, 90% (v/v) ethanol, 100% (v/v) ethanol, 100% (v/v) acetone and methanol: chloroform (2:3). These steps aimed to precipitate the soluble fibers, to remove small molecular weight components and to inactivate enzymes. The AIR obtained were dried overnight in a fume hood prior to extraction for immune glycome profiling.

### Cell Wall Glycome Profiling

Glycome analysis is an ELISA based technique which allows rapid analysis of polysaccharide epitopes found within solubilized cell wall fractions (Pattathil et al., 2010). AIR were sequential extracted with 50 mM CDTA, 4 M KOH and 1 μg/ml cellulase 5a (NZYTech). AIR (4 mg) was placed in 2 ml tubes and ball bearings were added into the sample before grinding in a Tissue Lyser at 50 Hz for 2 min. Then, 50 mM CDTA was added and ground for 20 min in the tissue lyser, followed by rocking of tube for 40 min and centrifuging at 3500 *g* for 15 min. The supernatant was kept as CDTA fraction, while the residues were then subjected to the next extracting reagent. The residues were extracted with 4 M KOH with 1% NaBH_4,_ giving the KOH fraction. Then, the residues were treated with 1 μg/ml cellulase in 20 mM Tris buffer pH 8.8 and incubated for 2 h at 37°C before centrifuging at 14000 rpm for 15 min. The supernatant was kept as cellulase fraction. The extracted cell wall fractions or supernatants from cell-separated samples were diluted 10 times before coating on the immunosorbent plates (Nunc) overnight at 4°C. Then, the plates were washed with tap water 9 times and blocked using 5% (w/v) non-fat dry milk in 10 mM PBS (M/PBS) for 2 h. After washing with tap water nine more times, 1:10 dilution of monoclonal antibodies in M/PBS (only 1:300 dilution for callose antibody) were added and incubated for 1.5 h. Each well of the plate contained a single type of antibody, and each antibody was done in duplicate wells. Forty antibodies were used in the analysis. The majority of them were rat monoclonal antibodies^1^, with the exception of anti-callose which was raised in mouse (BioSupplies, Australia). Following incubation with primary antibodies, the wells were washed with tap water nine times, then a 1:1000 dilution of secondary antibody in M/PBS (Anti-mouse IgG-HRP for the callose antibody and Anti-rat IgG-HRP for all others, both obtained from Invitrogen) was applied for 1 h. The plates were washed with tap water nine times, followed by the addition of the substrate to generate the signal. The substrate contained 1 M sodium acetate buffer pH 6.0, tetramethylbenzidine, 6% (v/v) hydrogen peroxide and distilled water with a ratio of 100:10:1:1000. The reaction was stopped by adding 2.5 M sulfuric acid, giving a yellow color. Binding strength of each antibody was determined by the absorbance at 450 nm via ELISA plate reader (Multiskan Fc microplate readers, Finland). Two fruit from each species were processed as biological replicates and each extract or supernatant analyzed on replicate wells.

### Data Analysis

For cell staining and immunofluorescence labeling (qualitative analysis), one microscopic image was chosen as a representative of the five images captured. For cell wall glycome profiling, the standard deviation was calculated using Microsoft Excel from two replicate experiments and coefficient of variation at <15% was set as an acceptable limit.

## Results

### Cell Separation and Cell Surface Staining of Banana and Mango Cells

**FIGURE 1:**
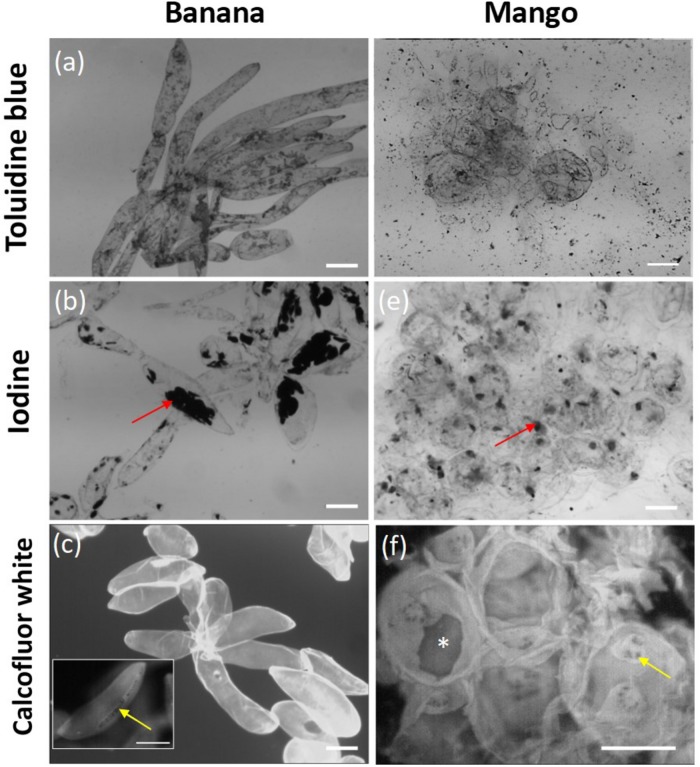
Micrographs of banana cells stained with toluidine blue **(a)**, iodine **(b)** and calcofluor white **(c)**; and mango cells stained with toluidine blue **(d)**, iodine **(e)** and calcofluor white **(f)**. Scale bar = 100 μm. Red arrows point to starch granules clearly visible in banana, yellow arrows point to the location of pit fields in strips in banana and round pits in mango. ^*^ Indicates tearing of the cell wall in mango fruit.

Parenchyma tissue from both fruits was ripe, soft, and the cells separated easily under mild stress. Tissue staining revealed some marked differences in the morphology of isolated cells (Figure 1). Banana tissue showed elongated, mostly intact cells which remained adherent by their apical tips, the outlines of the cells are visualized clearly with toluidine blue staining (Figure 1a). They contained several starch granules which stained strongly with iodine (Figure 1b). The intactness of the cell wall was confirmed by Calcofluor White staining (Figure 1c), which also revealed the presence of smalls holes, resembling pit fields (indicated by a yellow arrow on Figure 1c), organized on a narrow strip along the cell length. This pattern suggests that cells once adhered along this strip, but the adhesion was easily disrupted by minor stress (e.g., gentle scraping with spatula). In contrast, mango cells were rounder in shape (Figure 1d), and contained few starch granules (Figure 1e). Toluidine blue staining did not delineate the cells as clearly as for banana. The staining revealed oval structures on the surface of cells. We are not clear what those are, but could be the outline of large areas containing pit fields. Calcofluor White staining showed large sections of cell wall that appeared to have torn apart (indicated by ^*^ on Figure 1f), as well as brightly stained oval areas that contained abundant pit fields (indicated by an arrow on Figure 1f). The localization of pit fields in both banana and mango suggests that they may contribute to cell adhesion in these fruit.

**FIGURE 2:**
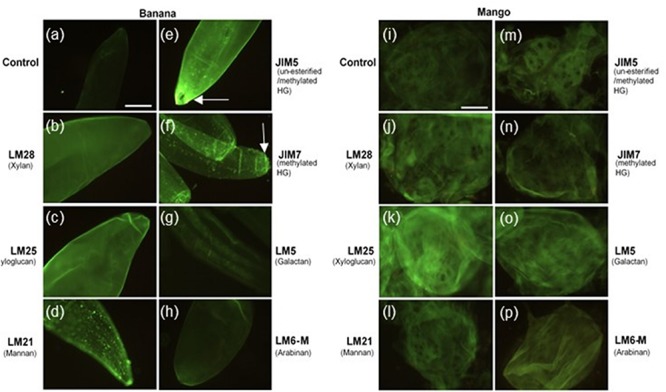
Banana **(a–h)** and mango **(i–p)** cells labeled with LM28, LM25, LM21, JIM5, JIM7, LM5 and LM6-M antibodies observed under fluorescence microscope equipped with blue epifluorescence. Scale bar = 50 μm. Arrows point to labeling at the tips of banana cells.

To investigate the distribution of cell wall polymers at the surface of cells in more detail, fruit tissue was labeled with seven monoclonal antibodies, which recognize different pectin and hemicellulose epitopes. As shown in Figure 2, banana cell walls showed strong and even distribution of hemicellulose epitopes, as labeled with LM28 (anti-xylan) and LM25 (anti-xyloglucan) antibodies. LM21 (anti-mannan) and JIM7 (anti-methyl esterified HG) showed punctate labeling throughout the cell wall. Bright fluorescence was detected with JIM5 labeling (anti-homogalacturonan), with the brightest labeling at the apex of the cells where cell adhesion was observed. Labeling of rhamnogalacturonan-I (RG-I) domains with LM5 (anti-galactan) and LM6 (anti-linear arabinan) was less intense, though a striated pattern could be discerned with LM5 labeling. Labeling of mango tissue showed a different pattern of labeling. The strongest labeling was observed with LM25 (anti-xyloglucan), followed by LM5 (anti-galactan) and LM8 (anti-xylan). No punctate labeling with JIM7 (anti-methylesterified HG) or LM21 (anti-mannan) was observed. Labeling with JIM5 antibody was weak, but stronger staining was observed on oval areas resembling the pit fields. In a similar way to banana, LM5 and LM6 labeling was not intense. The labeling patterns thus suggest a variation in surface properties of mango and banana cells.

### Atomic Force Microscopy

**FIGURE 3:**
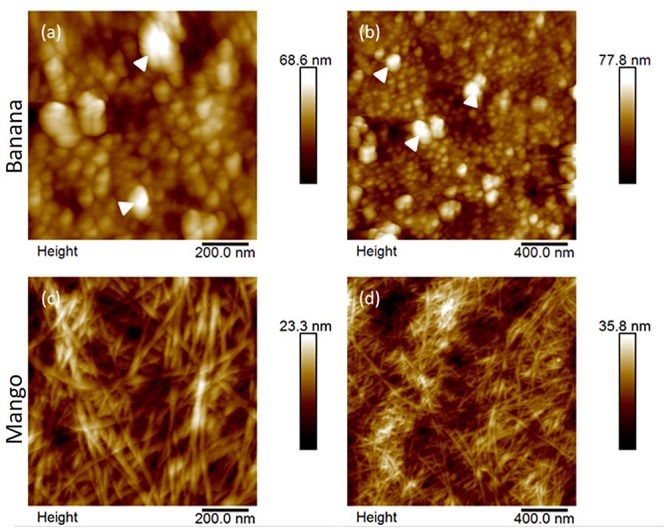
AFM height images of banana **(a,b)** and mango **(c,d)** cells at 1 μm (left) and 2 μm (right) scan sizes. Large aggregates on banana cell surfaces are indicated by white arrowheads. In contrast, fibrillar structures, attributed cellulose/hemicellulose are clearly visible in the mango cell wall.

The surface properties of shear separated banana and mango cells was evaluated with AFM. Only cells that had clearly separated (rather than ruptured) were scanned, to avoid observation of internal surfaces. Figure 3 shows representative pictures of cell surfaces, with marked differences in surface properties (height), with banana cells showing an amorphous texture with aggregates at the surface, which mask fibrous structures. This texture is attributed to middle lamella remnants, which did not solubilize during cell separation.

On the other hand, the surface of mango cells appeared cleaner, showing a clear network of microfibrils embedded in darker regions of matrix. This appearance suggests that a more advanced dissolution of the middle lamella had occurred in mango.

### Glycome Analysis of Cell Separation Supernatants and Cell Wall Extracts

We undertook the analysis of supernatant collected from separated cells as well as extracted polymers from AIR (Table 1).

**TABLE 1:** Cell wall glycome profiling of cell separation supernatants and fractions extracted from banana and mango AIR represented in heat map.

			**Banana**				**Mango**			
**Class**	**Epitope**	**Antibody**	**cell sep**	**CDTA**	**KOH**	**Cellulase**	**cell sep**	**CDTA**	**KOH**	**Cellulase**
Hemicellulose	Xylan	LM10	0.06 ± 0.00	0.05 ± 0.00	0.05 ± 0.00	0.05 ± 0.00	0.07 ± 0.00	0.05 ± 0.00	0.05 ± 0.01	0.05 ± 0.01
	Xylan/arabinoxylan	LM11	0.06 ± 0.01	0.05 ± 0.00	0.07 ± 0.00	0.08 ± 0.01	0.08 ± 0.00	0.07 ± 0.01	0.08 ± 0.00	0.09 ± 0.00
	Grass xylan	LM12	1.02 ± 0.05	0.08 ± 0.01	0.06 ± 0.01	0.05 ± 0.00	0.06 ± 0.00	0.06 ± 0.00	0.05 ± 0.00	0.05 ± 0.00
	Glucuronoxylan	LM28	0.09 ± 0.00	0.06 ± 0.00	0.35 ± 0.03	0.68 ± 0.02	0.12 ± 0.02	0.17 ± 0.01	0.29 ± 0.00	0.42 ± 0.09
	Xyloglucan	LM15	0.16 ± 0.01	0.09 ± 0.05	1.44 ± 0.01	0.12 ± 0.01	0.45 ± 0.02	1.86 ± 0.02	1.67 ± 0.02	1.17 ± 0.04
	Xyloglucan	LM24	0.00 ± 0.00	0.04 ± 0.00	0.05 ± 0.00	0.05 ± 0.00	0.13 ± 0.02	1.35 ± 0.10	0.08 ± 0.00	0.07 ± 0.00
	Xyloglucan	LM25	0.60 ± 0.25	0.08 ± 0.02	1.58 ± 0.04	0.45 ± 0.01	1.04 ± 0.01	1.84 ± 0.01	1.76 ± 0.05	1.60 ± 0.06
	Mannan	LM21	1.35 ± 0.20	1.72 ± 0.03	0.77 ± 0.01	0.15 ± 0.01	0.08 ± 0.01	1.65 ± 0.00	0.12 ± 0.01	0.11 ± 0.02
	Mannan	LM22	0.00 ± 0.00	0.05 ± 0.00	0.05 ± 0.00	0.05 ± 0.00	0.08 ± 0.00	0.05 ± 0.00	0.05 ± 0.00	0.06 ± 0.00
	Mannan	LM30	0.09 ± 0.01	0.05 ± 0.00	0.05 ± 0.00	0.05 ± 0.00	0.19 ± 0.00	0.12 ± 0.01	0.09 ± 0.00	0.08 ± 0.01
Pectins	HGA	LM7	0.08 ± 0.01	0.05 ± 0.00	0.09 ± 0.01	0.11 ± 0.00	0.10 ± 0.01	0.11 ± 0.02	0.08 ± 0.01	0.13 ± 0.02
	HGA	LM18	1.30 ± 0.00	0.10 ± 0.00	0.13 ± 0.00	0.22 ± 0.01	0.73 ± 0.05	1.00 ± 0.01	0.32 ± 0.02	0.89 ± 0.02
	HGA	LM19	1.15 ± 0.13	0.17 ± 0.00	0.53 ± 0.04	1.17 ± 0.07	1.12 ± 0.26	1.27 ± 0.04	0.66 ± 0.04	1.55 ± 0.06
	HGA	LM20	0.59 ± 0.05	1.27 ± 0.11	0.05 ± 0.00	0.05 ± 0.00	1.14 ± 0.06	1.84 ± 0.05	0.05 ± 0.00	0.05 ± 0.00
	HGA	JIM5	1.52 ± 0.10	0.97 ± 0.13	0.32 ± 0.01	0.32 ± 0.02	1.36 ± 0.01	2.04 ± 0.06	0.33 ± 0.00	0.78 ± 0.00
	HGA	JIM7	1.23 ± 0.12	1.22 ± 0.06	0.05 ± 0.01	0.05 ± 0.00	1.50 ± 0.02	1.80 ± 0.03	0.06 ± 0.01	0.06 ± 0.01
	Galactan	LM5	0.20 ± 0.02	0.05 ± 0.00	0.11 ± 0.01	0.22 ± 0.02	0.19 ± 0.00	1.87 ± 0.02	1.62 ± 0.05	1.76 ± 0.01
	Branched galactan	LM26	0.08 ± 0.00	0.05 ± 0.00	0.07 ± 0.00	0.08 ± 0.00	0.49 ± 0.09	0.31 ± 0.00	0.36 ± 0.01	0.29 ± 0.02
	Arabinan	LM6-M	0.42 ± 0.00	0.08 ± 0.00	0.18 ± 0.00	0.47 ± 0.03	0.55 ± 0.02	1.03 ± 0.07	0.82 ± 0.03	1.10 ± 0.03
	Linear arabinan	LM13	0.07 ± 0.04	0.05 ± 0.00	0.10 ± 0.04	0.08 ± 0.01	0.18 ± 0.01	0.07 ± 0.00	0.10 ± 0.00	0.32 ± 0.02
	Processed arabinan	LM16	0.11 ± 0.00	0.05 ± 0.00	0.07 ± 0.00	0.12 ± 0.00	0.36 ± 0.01	0.28 ± 0.02	0.44 ± 0.05	0.43 ± 0.02
	Xylogalacturonan	LM8	0.10 ± 0.00	0.07 ± 0.00	0.07 ± 0.01	0.08 ± 0.00	0.09 ± 0.00	0.04 ± 0.01	0.09 ± 0.01	0.13 ± 0.02
Glycoproteins	AGP	LM2	0.73 ± 0.00	0.22 ± 0.02	0.41 ± 0.01	0.31 ± 0.04	0.86 ± 0.00	0.67 ± 0.10	0.34 ± 0.00	0.31 ± 0.01
	AGP	LM14	0.38 ± 0.11	0.10 ± 0.00	0.14 ± 0.00	0.11 ± 0.00	0.13 ± 0.00	0.15 ± 0.01	0.08 ± 0.00	0.09 ± 0.01
	AGP	JIM4	0.12 ± 0.07	0.05 ± 0.00	0.05 ± 0.00	0.05 ± 0.00	0.00 ± 0.00	0.06 ± 0.00	0.05 ± 0.00	0.06 ± 0.00
	AGP	JIM8	0.07 ± 0.05	0.04 ± 0.00	0.06 ± 0.00	0.07 ± 0.01	0.17 ± 0.00	0.06 ± 0.00	0.07 ± 0.00	0.08 ± 0.01
	AGP	JIM13	0.08 ± 0.00	0.05 ± 0.00	0.06 ± 0.02	0.05 ± 0.01	0.80 ± 0.02	1.09 ± 0.08	1.18 ± 0.01	0.77 ± 0.03
	AGP	JIM15	0.08 ± 0.00	0.06 ± 0.01	0.13 ± 0.01	0.14 ± 0.00	0.12 ± 0.02	0.05 ± 0.01	0.12 ± 0.01	0.12 ± 0.02
	AGP	JIM16	0.74 ± 0.00	0.07 ± 0.00	0.06 ± 0.00	0.05 ± 0.00	0.75 ± 0.00	0.95 ± 0.06	0.06 ± 0.01	0.05 ± 0.01
	AGP	MAC207	0.49 ± 0.00	0.10 ± 0.00	0.16 ± 0.00	0.12 ± 0.01	0.15 ± 0.04	0.20 ± 0.01	0.09 ± 0.00	0.08 ± 0.01
	Extensin	LM1	0.40 ± 0.09	0.07 ± 0.00	1.17 ± 0.00	0.64 ± 0.02	0.32 ± 0.00	0.35 ± 0.03	1.44 ± 0.03	1.22 ± 0.04
	Extensin	JIM11	0.14 ± 0.08	0.05 ± 0.00	0.05 ± 0.00	0.05 ± 0.00	0.51 ± 0.01	0.56 ± 0.03	1.13 ± 0.02	0.95 ± 0.02
	Extensin	JIM12	0.11 ± 0.01	0.09 ± 0.05	0.57 ± 0.02	0.20 ± 0.02	0.08 ± 0.06	0.06 ± 0.00	0.93 ± 0.02	0.70 ± 0.03
	Extensin	JIM19	0.12 ± 0.00	0.04 ± 0.00	0.05 ± 0.00	0.05 ± 0.00	0.00 ± 0.00	0.05 ± 0.00	0.05 ± 0.00	0.05 ± 0.00
	Extensin	JIM20	0.28 ± 0.01	0.06 ± 0.00	0.43 ± 0.02	0.17 ± 0.00	0.36 ± 0.00	0.36 ± 0.04	1.39 ± 0.00	1.16 ± 0.03
Callose			0.00 ± 0.00	0.06 ± 0.00	0.29 ± 0.02	0.12 ± 0.03	0.00 ± 0.00	0.11 ± 0.01	0.81 ± 0.01	0.48 ± 0.02

*The strength of ELISA signals (mean of two replicate experiments, A_450_ ± standard deviation) was shown in a white to red scale with deepest color representing highest relative absorbance.*

When looking at epitopes solubilized during cell separation, the glycome profiles appeared similar in banana and mango samples. In both cases, pectin epitopes detected with LM18, LM19, LM20, JIM5, and JIM7 had the highest relative abundance, indicating solubilisation of both methylated and un-methylated HG into the cell separation supernatant. Pectin arabinan, but not galactan, was also detected in the soluble fraction in both fruits. The substituted xyloglucan epitope recognized by LM25 (xyloglucan with XLLG, XXLG, and XXXG motif, where L and G show different substitutions on the xyloglucan backbone) was also detected in both fruit supernatants. The key difference to highlight between the two fruits was the presence of mannan (recognized by the LM21 antibody) and ferulated xylan (recognized by the LM12 antibody) in banana cell separation supernatant, but not in mango. This analysis confirms the presence of mannan at the surface of banana cells, some of which solubilizes during cell separation. It must be noted that not steps were taken to inactivate enzymes during the cell separation experiments, as most procedures used to inactivate enzymes would likely impact on cell separation and polymer solubilisation. The role of endogenous enzymes in texture perception is needs further investigation. Recently, PME activity during oral processing of tomato was observed (Rabiti et al., 2018).

Sequential extractions with CDTA, KOH and cellulase extract cell wall polymers from AIR. In general, the level of soluble epitopes were higher in mango compared to banana. In particular, CDTA solubilized more HG and xyloglucan epitopes from mango AIR compared to banana. Mannan was solubilized from both fruits with CDTA, suggesting it is easily extractable. The LM5 epitope was very abundant in all mango fractions, but only detected minor levels detected in banana. Branched galactan epitopes detected by LM26 were detected at low levels in all mango fractions, but not in banana. The relative abundance of AGPs and extensins was higher in mango compared to banana for most antibodies used. Glycome analysis allows rapid analysis of polysaccharide epitopes found within solubilized cell wall fractions (Pattathil et al., 2010). However, it does not allow quantitative determination of the polymers.

### Bulk Rheology

**FIGURE 4:**
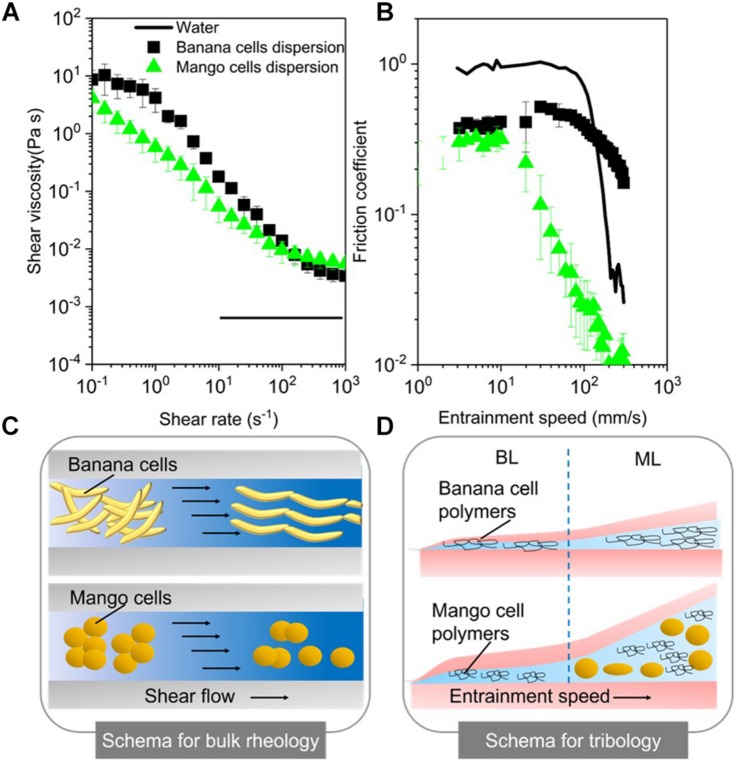
Apparent viscosities as a function of shear rates **(A)** and friction coefficients as a function of entrainment speeds **(B)** for mango and banana cell suspensions with respective schematics displayed for rheology **(C)** and tribology phenomena (BL, Boundary lubrication regime; ML, mixed lubrication regime) **(D)**. MilliQ water was used as a control in both rheology and tribology experiments. Error bars represent the standard deviation of at least three measurements.

Figure 4A shows that the aqueous suspensions of both mango and banana cells display a clear shear thinning behavior with apparent viscosities showing a three-orders of magnitude reduction as a function of shear rate within the experimental window. The observed shear thinning behavior of these cell suspensions might be attributed to the shear flow-induced disruption of those aggregates of banana or mango cells into individual cells that were aligning in the direction of flow as shown in the schema (Figure 4C).

Noteworthy, banana cell suspension showed a definite zero shear rate limiting viscosity at 10 Pa.s and a second Newtonian plateau at 3 × 10^–3^ Pa.s. On the other hand, mango cell suspension exhibited extreme shear thinning behavior, with plateau values not observed until shear rate of 100 s^–1^. Of more relevance here is the fact that both the systems showed very similar viscosities (0.05 Pa.s) (*p* > 0.05) at orally relevant shear rate of 50 s^–1^ (Ong et al., 2018) and also similar terminal viscosities at and above 100 s^–1^. Young’s modulus of plant cells measured using AFM probe may range from 100 kPa to 1 MPa (Radotić et al., 2012; Zdunek and Kurenda, 2013). Even at the highest shear rates (1000 s^–1^) used in this study, the shear stress on the cells imposed by the carrier fluid cannot be larger than 10 Pa. Hence, both the systems can be hypothesized to retain intact cells after shearing, as schematically shown in Figure 4C, providing structural aspects with higher resistance to flow as compared to water.

### Soft Tribology

The lubrication performance of mango and banana cells suspensions are shown in Figure 4B, where the friction coefficient (μ) is plotted against entrainment speeds. A plot of μ versus entrainment speeds for Milli-Q water is also shown for comparison purposes. The boundary lubrication regime is commonly found at the lowest entrainment speeds (≥10 mm s^–^1) and is characterized by relatively high μ values that show no dependence on the speed (dry friction). In Figure 4B, the boundary regime is clearly observed for both mango and banana cell suspensions. Irrespective of the fruit type, both cell suspensions showed similar μ values (*p* > 0.05) in the boundary lubrication regime, being significantly lower than water (Figure 4B). This indicates lubricating behavior.

Considering the size of the cells (100–150 μm in diameter), it is highly unlikely for either of the cell types to enter into the contact zone. Even if they would have entered the contact zone, they would have flattened (Sarkar et al., 2017; Torres et al., 2018) or ruptured owing to the high pressures within the confinement. Therefore, such reduction of μ values in boundary regime suggests that it was not due to entrainment of intact cells (if any remaining), but due to the soluble polymers in the continuous phase.

These soluble polymers were plausibly adsorbing to the surfaces and forming films of few molecules thickness (schematically shown in Figure 4D) and reducing μ as compared to that of water (*p* < 0.05). This remarkable boundary lubrication behavior is unlike the behavior of starch granule ghosts observed in a previous report (Zhang et al., 2017), where their boundary lubrication profiles were close to water due to non-adsorbing starch polymers being present in the continuous phase.

As the entrainment speed increased (≥10 mm s^–1^), the curves showed the mixed lubrication regime with decrease of μ values. The reduction in μ in this regime is associated with partial separation of the contact surfaces by a discontinuous layer of lubricant (Sarkar et al., 2019), where pressure is borne both by the lubricant and the surfaces. As can be observed in Figure 4B, it is in the mixed regime, where the cell type showed distinctiveness in their lubrication properties. In particular, mango cells with nearly spherical appearance (around 150 μm size) showed a much faster onset of mixed lubrication regime (≥10 mm s^–1^) with dramatic reduction of μ (μ < 0.05) in orally relevant speeds (50 mm s^–1^). In case of ellipsoidal shaped banana cells, the boundary regime was extended until 100 mm s^–1^ (Figure 4B), which suggests that there is limited likelihood that the banana cells were entering the contact at orally relevant speeds. In this case, the carbohydrate polymers solubilized during cell separation could have an impact on the rheology and tribology behavior of cell suspensions.

## Discussion

Cell separation due to the solubilisation of the middle lamella polymers, as well as primary cell wall disassembly are suggested to contribute to the textural perception of ripe fruits. The results of this study suggest that banana cell walls disassemble in a different way to mango cell walls during ripening-associated softening. Banana cells separate very easily under stress but remain apparently intact suggesting weak middle lamella but stronger primary walls. According to the AFM, banana cells seem to retain aggregated material at the surface, proposed here to be middle lamella remnants. These aggregate structures resemble those observed using AFM of extracted pectins from unripe strawberry (Paniagua et al., 2014), but this is the first time they are observed directly *in muro*. Immunofluorescence microscopy suggested that these aggregates be methylesterified HG or mannan, which appeared as punctate labeling on the surface of cells. Galactan also appears to have a distinct pattern of labeling at the surface that suggests aggregation at the cell surface. Furthermore, glycome profiling confirmed the presence of pectins and mannans in the supernatant of separated banana cells. Mannans have been shown to be major components of banana cells walls, with relatively good solubility (Shiga et al., 2017). Isolated mannans form weak gels that break and deform easily under strain (Ben-Zion and Nussinovitch, 1997). This property may be very useful for banana to keep weak adhesion between cells that is easily disrupted using mechanical force, without need for enzymatic breakdown. It is not clear whether this cell separation behavior is in some way related to seed dispersal, or whether it has been selected in by human breeding. Mannans and other hemicelluloses have been suggested to have a role in cell adhesion in ripening tomato fruit (Ordaz-Ortiz et al., 2009). The presence of ferulated xylan in the cell separation supernatant is unexpected, since they are normally extracted from insoluble cell wall fractions (Schendel et al., 2016; Ruthes et al., 2017) and localized in pericarp and aleurone layers of hardening cell walls in developing maize grains (Chateigner-Boutin et al., 2016). Their presence has not been show in banana fruit and their role needs further investigation.

The intactness of banana cells, their size and shape (high aspect ratio (length/diameter) i.e., 2-4:1) decrease the chances of entrainment between oral surfaces i.e., tongue and palate, translating into possible astringency perception. Indeed, the banana cells were excluded from entering the contact zone as schematically shown in Figure 4D and thus resulted in some degree of asperity, as cells did not reduce friction. The aggregates of mango or banana cells observed in Figure 1 most likely represented a larger effective volume fraction than that of their constituent individual cells and consequently, generated increased viscosity values at low shear rates (10^–1^ s^–1^) (Genovese, 2012; Moelants et al., 2014). Banana cells also remain intact during chewing and gastro-intestinal digestion (Low et al., 2015; Chu et al., 2017) and this resilience was apparent in the friction experiments where banana cells did not break at higher shear rates. The resilience could be explained by higher deformability or higher mechanical strength. Both could result in less rupturing. Further AFM experiments that measure mechanical strength of cell walls are required to assess the properties of intact banana cells. The health implications of intact cell walls are emerging. Banana cells were shown to be less susceptible to microbiota fermentation compared to mango (Low et al., 2015). Meanwhile, polysaccharides solubilized from banana pulp, including mannans, pectins and AGPs were shown to elicit immunomodulatory responses of benefit to gut health (Shiga et al., 2017). Pectins and mannans were found in the cell separation supernatants confirming their easy of solubility.

Mango cells, on the other hand, both separated and ruptured. The surfaces of separated cells observed with AFM suggested more pronounced disassembly of middle lamella and cell walls in those regions. However, the higher propensity to tearing of mango cells suggests strong cell adhesion in other regions, likely to be associated with pit fields. The physical, chemical and biological changes to mango cell walls during ripening were elegantly studied using a range of methods (Cárdenas-Pérez et al., 2018). High PME and endo-PG activity at later stages of ripening led to increased solubility of pectin, shorter and less organized polymers (as seen by AFM), and mechanically weaker cell walls. These molecular changes were correlated to softer textures at the tissue scale (Cárdenas-Pérez et al., 2018). These observations are corroborated here, as mango cell walls appeared deformable under low shear leading to form a layer that lowered friction in the tribology experiments. The main polymers solubilized during cell separation of mango cells were mainly pectins and xyloglucans, while mannan was only solubilized with chemical treatment. Their solubilisation and cell wall disassembly in general is explained by endogenous cell wall enzyme activities during ripening including PME, endo-PG, PL and XTH (Chourasia et al., 2006, 2008). The solubilized material that may also contribute to the faster onset of the mixed lubrication regime in mango cells, which can be interpreted as a smooth and slippery mouthfeel.

Bulk rheology results suggest that mango and banana cell suspensions have similar bulk viscosity at orally relevant shear rates and hence might be extrapolated to have similar “oral thickness” perception in the initial stages of oral processing. But the significant differences in their bio-lubrication behavior may explain their different textural attributes in later stages of processing that include friction between oral surfaces (e.g., tongue and palate). For instance, the lower friction between the soft contact surfaces in this tribological experiments (emulating the tongue and oral palate) in case of the mango cells is associated with incorporation of mango cells between these contact surfaces at orally relevant speeds (Figure 4). Such lower friction might be reflected as “smooth” sensory perception after oral processing of mangoes as the tongue can be hypothesized to be separated from the oral palate by a thin layer of mango cells and not rubbing against the oral palate. On the other hand, in case of banana cells, they were not entering the contact (Figure 4D), which might be interpreted in real life oral processing as tongue was rubbing against the oral palate in absence of any cells resulting in increased friction, which might be reflected as “rough” or “astringent perception.” The combination of rheology and tribology with cell wall analysis used in this study for the first time offers a unique approach to gain mechanistic understanding of the contribution of cells and cell wall polymers to texture perception of ripe fruits. Furthermore, such knowledge can be also used to quantitatively understand the mechanisms behind sensory mouthfeel in fruits as well as in semi-solid foods, such as fruit purees and fruit-rich baby foods where bulk rheology alone is not sufficient to mechanistically explain the surface interactions occurring at later stages of oral processing. Future studies need to be conducted with various concentrations of cell suspensions to clearly investigate the effect of volume fraction of cells, the elastic modulus of cells, the role of saliva and the interaction of saliva with both cells and cell wall polymers. Instrumental studies should be supported with quantitative sensory analysis to examine instrument-mouthfeel correlations.

## Data Availability

The raw data supporting the conclusions of this manuscript will be made available by the authors, without undue reservation, to any qualified researcher.

## Author Contributions

CO and YB-A conceived the research project. GR and SA performed the microscopy and glycome experiments. EA-R performed the bulk rheology and tribology experiments. HL performed the AFM experiments under the supervision of CO and SC. GR, SA, EA-R, AS, and CO analyzed the data. GR, CO, and AS wrote the manuscript. YB-A and JPK critically reviewed and finalized the manuscript.

## Conflict of Interest Statement

The authors declare that the research was conducted in the absence of any commercial or financial relationships that could be construed as a potential conflict of interest.

## Footnotes

1 http://www.plants.leeds.ac.uk/pk/pdf/JPKab05.pdf
